# Supplementary material for: BCG epidemiology supports its protection against COVID-19? A word of caution
Source: PLoS One. 2020 Oct 7;15(10):e0240203. doi: 10.1371/journal.pone.0240203 (PMC7540851; doi:10.1371/journal.pone.0240203)
Supplement: Attachment — Submitted filename: ResponseToReviewers_BCG COVID-19 R1 Letterhead.doc [file pone.0240203.s002.doc]

www.bcm.edu

Department of Pediatrics

One Baylor Plaza

Houston, Texas 77030-3411

**Gastroenterology, Hepatology and Nutrition**

**Mission Statement**

To strive for outstanding and compassionate patient care

6701 Fannin Street

MC CC1010.00

Houston, TX 77030-2299

through evidence-based and expert practice achieved through

research and education

## FACULTY

**Richard Kellermayer**

**Associate Professor of Pediatrics**

**Baylor College of Medicine**

**Texas Children's Hospital**

Benjamin L. Shneider, M.D.

Chief of Section

Anthony O. Anani, MD, MBA

Beth A. Carter, M.D.

Eric H. Chiou, M.D.

Andrew Chu, M.D.

Bruno P. Chumpitazi, M.D., MPH

Douglas S. Fishman, M.D., FASGE

Donna K. Garner, RN, MSN, PNP

Caroyl Gilbert, RN, MSN, PNP

G. S. Gopalakrishna, M.D.

Sanjiv Harpavat, M.D., Ph.D.

Paula M. Hertel, M.D.

Ryan W. Himes, M.D.

John M. Hollier, M.D.

Faith D. Ihekweazu, M.D.

Craig L. Jensen, M.D.

Lina B. Karam, M.D.

Richard Kellermayer, M.D., Ph.D.

Kristi L. King, M.P.H., R.D., L.D.

Seiji Kitagawa, M.D.

Daniel H. Leung, M.D.

Jennifer M. Maupin, MS, RN, CPNP

Carmen Mikhail, Ph.D.

Tamir Miloh, MD

Kathleen J. Motil, M.D., Ph.D.

Krupa Mysore, M.D.

Anthony P. Olivé, M.D.

Yen Pham, M.D.

Sarah M. Phillips, M.S., R.D.

Priya Raj, M.D., M.S.

Carol A. Redel, MNS, M.D.

Ann O. Scheimann, M.D.

Vernisha Y. Shepard, M.Ed., LPC

Robert J. Shulman, M.D.

Mary Elizabeth Tessier, MD

Sundararajah Thevananther, Ph.D.

Nicole Triggs,MSN,APRN,CPNP,CPN

Kristin L. Van Buren, M.D.

Bryan S. Vartabedian, M.D.

Seema Mehta Walsh, M.D.

Allyson E. Wyatt, M.D.

##### TELEPHONE

**Office:**

Office Fax:	832-825-3633

Clinic:	832-822-3131

Clinic Fax:	832-825-4131

Appointments:	832-822-2778

OUTREACH CLINICS

Sugar Land	281-494-7010

Cy-Fair	281-469-4688

Clearlake	281-282-1900

West Campus	832-227-1440

Woodlands	936-321-0808

**Response to the Reviewers**

The time and expertise of the reviewers is greatly appreciated. We responded to the valuable recommendations point-by-point below. The corresponding changes in the manuscript are highlighted with Word ‘track changes’. The modifications have greatly improved the quality of our manuscript. In general, we would like to underscore that our work is intended to demonstrate that the currently available epidemiological data on COVID-19 can not be used to make any reliable clinical prediction in regards to prevention or treatment against SARS-CoV-2, especially if examined as death/million. We wished to deliver this message in the most scientific way possible in order to effectively reach the biomedical research community. Hence, the “word of caution” in our title. The title has also been modified to more precisely cover our findings and message, which pertains to BCG epidemiology, and does NOT intend to evaluate the biologic evidence for BCG vaccination as an acute (i.e. short lived) protectant against COVID-19. We feel that PLOS ONE is and outstanding means to communicate this message. We added Giedrius Trakimas to our author list and performed additional analyses to more strongly support our conclusions.

Sincerely,

Richard Kellermayer M.D., Ph.D.

Associate Professor

Director, Pediatric Inflammatory Bowel Disease Program

Baylor College of Medicine, Texas Children's Hospital

6621 Fannin St., CC1010.00

Houston, TX 77030-2399

Voice: 713-798-0319; Fax: 713-798-0338

Reviewer #1: The article from Richard Kellermayer’s group presents a critical analysis of the recent publication by Miller et al 2020 ref. [15] and proposes an original approach to establish a representation of the daily rates of COVID-19 case fatality (i.e. Death Per Case /Days of the endemic [dpc/d]). However, only strictly identical groups (age/sex proportion, living conditions, COVID diagnosis) could be strictly compared and would better answer the question of the effect of the BCG vaccine on current acute epidemic infection; could you address this question by adding an analysis of equivalent groups (for ex. in urban conditions)? Furthermore, to support the article statements, it is necessary to discuss whether the people who have benefited from BCG vaccination or not are really comparable (age/sex proportion, living conditions, COVID diagnosis), and otherwise to highlight the differences observed. Finally, he possible bias of the chosen representation is not discussed.

Response: Our general response partly addresses this recommendation of the distinguished Reviewer. We absolutely agree that if reliable and sound epidemiologic data were available, stricter analyses incorporating as many variables as possible would be desired. In the meantime, we have now performed multiple regression modeling and incorporated additional variables such as the recommended ‘urban living’ as predictors into our models. Our main conclusion has not been significantly modified by these analyses. We do highlight the arbitrary selection bias not only in respect to BCG vaccination (as the essence of our critique manuscript), but in regards to our ‘historical colonization status’ variable.

In the abstract and elsewhere after, it’s supported that “the use of personal protective equipment (PPE) are the only epidemiologic measures” while the facts exemplified may demonstrate opposite situations: please add also that PPE in our day-to-day lives are efficient when associated with frequent and proper hand washing.

Response: We decided to omit the discussions about PPE in respect to COVID-19, since that is not the focus of our work.

There is too much citations from media and general press (e.g. BBC, YouTube…) in the R. Kellermayer’s article that could be replaced with scientific short communications or opinion views. A lot of references citations of key publications are lacking concerning the current epidemic of COVID-19 both in introduction and in discussion part (for example in the introduction: Lescure FX (https://doi.org/10.1016/S1473-3099(20)30237-1) in parallel to ref. [3] and He X https://www.nature.com/articles/s41591-020-0869-5 in parallel to ref. [4, 5]).

Response: Thank you for the recommendations, we have incorporated the references. In the meantime, we would like to emphasize that the focus of our work is the critique of worldwide epidemiology data, not a comprehensive review of COVID-19 pathogenesis.

One point that particularly deserves to be developed in this article is the explanation of the interest especially in the BCG vaccination (even extension of use, see thereafter) in the context of COVID-19 epidemic: please explain the scientific rational.

Some evaluations in children of a BCG vaccine cross-protection against several types of microbial or fungal infection were published yet (doi:10.1016/j.cmi.2019.04.020 ; doi:10.3389/fimmu.2019.02806) and these studies are not mentioned or discussed. Likewise, there is no reference to the phase III clinical trials which have recently started (Holland (NCT04328441); Australia (NCT04327206)) to answer the question of the prophylactic effect of BCG vaccine on COVID-19, dedicated to health care, aiming at reducing the symptomatology and considered as an emergency measure and not a lasting solution.

In addition, many applications for prophylactic purposes currently take advantage of the anti-inflammatory response induced by this BCG vaccination, e.g. for type 1 diabetes, bladder cancer, or autoimmune diseases. This rational for use should also be discussed.

Response: We have included a brief summary of the plausible biologic basis for BCG vaccination protection, and referenced a comprehensive and freely accessible web page based review on the topic in our discussion. We also included into our introduction the number of clinical trials registered in clinicaltrials.gov in June. However, our focus is to emphasize the highly biased and interdependent nature of arbitrary predictors of COVID-19 epidemiology, especially in review of the unreliable nature of country based case and death reporting.

Reviewer #2: The authors explored the association between daily rates of COVID-19 case fatality and the presence of universal BCG vaccination.

At the time of submission of their manuscript, there was one preprint reporting a correlation between universal BCG vaccination policy and reduced morbidity and mortality for COVID-19, which has not published yet after peer-review (at least, it is not listed in pubmed as for today, the 2nd of May).

The current analysis defined BCG vaccination data based on the same database as the preprint they refer to. For the death rate data, they used the one reported on April 3rd (instead of March 31st) and they did not exclude countries with less than one million inhabitants.

They analyzed the association between the death rate or the Death Per Case (or case fatality rate)/Days from onset (dpc/d) to account for testing and time bias, and BCG vaccination. They also compared Netherlands, Spain, United Kingdom, France, Belgium, Portugal, and Germany (they called Modern "Colonial Era" countries to colonize America and Africa) to others.

They showed that there is significantly less death/million when BCG was introduced before 1980, but it is not the case when Death Per Case/Days of endemic (dpc/d) is considered. Finally, they reported that dpc/d is significantly higher in Modern Era Colonizer countries compared to others.

MAJOR RECOMMANDATIONS

The choice of 1980 has a cut off for having BCG was defined arbitrary. The authors state that “BCG would have affected the population of a country 40 years old and above [i.e. the population with increased vulnerability towards the SARS-CoV-2 infection]”. In studies reporting a non specific effect of live attenuated vaccines (such as BCG, oral polio, measles or smallpox), like the one published by Christine Benn and Peter Aaby, the heterologous protection against all cause morbidity and mortality is long-lasting, but as compared to the transient immediate defense mediated by the innate effector response. It is not as long-lasting as adaptive immune memory, and in no way it lasts 40 years. Moreover, the non potential beneficial non specific effect of BCG might be modulated with time, and other vaccines for instance. Thus, the authors could focus their analysis on a younger population, rather than the entire population of each of the Top 68 countries in terms of covid-19 induced death, or covid-19 severity.

Response: Thank you for the insightful observations and recommendations. We absolutely agree with this Reviewer that there is no biologic basis for newborn/baby age BCG vaccination to protect against COVID-19 infection and/or morbidity in the elderly. We have now performed analyses on an additional (May 15^th^) dataset with multiple regression modeling, including median age as a predictor. We have also added a recent publication as reference (37) that contradicts newborn BCG effects on COVID-19, even in young adults around the age of 40. Our main conclusion is the unreliable nature of worldwide COVID-19 epidemiologic data and that researchers should consequently avoid, or highly critically perform detailed analyses of that.

The year of introduction of BCG vaccination can be one variable to study. However, the authors are certainly aware of the heterogeneity of the vaccine coverage depending on the country. Whether BCG vaccination is mandatory or not can result in different BCG vaccine coverage. In addition, the vaccine schedules and strains of BCG with different immunogenicity can differ between countries for instance. This should ideally be taken into account because it can be confounding, or at least be discussed by the authors.

Response: We would like to repeatedly emphasize the main point of our work, which underscores the unreliable nature of COVID-19 worldwide data. We trust that the added analyses and more clear discussion of those along with a stronger message to the epidemiologist community makes it understandable that we decided not to perform the recommended studies on the heterogeneity of the vaccine coverage, depending on the country.

MINOR RECOMMANDATIONS

Please update the introduction and discussion sections with the very recent literature about:

-The presence of viral RNA found in plasma and urine, and the successfully isolation of infectious viruses from these compartments (as for the presence of infectious particles in feces, there are inherent technical difficulties to isolate virus from feces because of the high risk of contamination of cell cultures with other microrganisms than the virus of interest)

-the transmission through aerosols

-the pathogenesis of SARS-CoV-2 (and not SARS-CoV) in non human primates, including the comparison between n=2 old and n=2 young animals

-there are currently several preprints on observationnal studies and the analysis of the association between BCG vaccination and covid-19, with positive, negative or no correlation

-there are now more than one trial to evaluate whether BCG could reduce the susceptibility to SARS-CoV2 infection and severity of covid-19

Please include a paragraph on the hypothesis as to how BCG could protect, or not, or even worsen the susceptibility to SARS-CoV2 infection and severity of covid-19, with few words on the concept of non specific effect of vaccines and on the new paradigm of trained immunity.

The quality of the figures is too low.

Response: We repeatedly hope that the added analyses, additional references and more clear discussion of those along with a stronger message to the epidemiologist community is acceptable to this Reviewer without us following all the minor recommendations. We wished to be as concise as possible and not elaborate more on viral transmission and animal modeling in respect to SARS-CoV-2 pathogenesis. We did include a paragraph referencing a rather recent comprehensive review on the plausible connections between BCG vaccination, and off target viral infections, including COVID-19. We also excluded our figures during the major revision of our work.
