# Supplementary material for: BCG epidemiology supports its protection against COVID-19? A word of caution
Source: PLoS One. 2020 Oct 7;15(10):e0240203. doi: 10.1371/journal.pone.0240203 (PMC7540851; doi:10.1371/journal.pone.0240203)
Supplement: Attachment — Submitted filename: ResponseToReviewers_BCG COVID-19 R2 Letterhead.doc [file pone.0240203.s003.doc]

www.bcm.edu

Department of Pediatrics

One Baylor Plaza

Houston, Texas 77030-3411

**Gastroenterology, Hepatology and Nutrition**

**Mission Statement**

To strive for outstanding and compassionate patient care

6701 Fannin Street

MC CC1010.00

Houston, TX 77030-2299

through evidence-based and expert practice achieved through

research and education

## FACULTY

**Richard Kellermayer**

**Associate Professor of Pediatrics**

**Baylor College of Medicine**

**Texas Children's Hospital**

Benjamin L. Shneider, M.D.

Chief of Section

Anthony O. Anani, MD, MBA

Beth A. Carter, M.D.

Eric H. Chiou, M.D.

Andrew Chu, M.D.

Bruno P. Chumpitazi, M.D., MPH

Douglas S. Fishman, M.D., FASGE

Donna K. Garner, RN, MSN, PNP

Caroyl Gilbert, RN, MSN, PNP

G. S. Gopalakrishna, M.D.

Sanjiv Harpavat, M.D., Ph.D.

Paula M. Hertel, M.D.

Ryan W. Himes, M.D.

John M. Hollier, M.D.

Faith D. Ihekweazu, M.D.

Craig L. Jensen, M.D.

Lina B. Karam, M.D.

Richard Kellermayer, M.D., Ph.D.

Kristi L. King, M.P.H., R.D., L.D.

Seiji Kitagawa, M.D.

Daniel H. Leung, M.D.

Jennifer M. Maupin, MS, RN, CPNP

Carmen Mikhail, Ph.D.

Tamir Miloh, MD

Kathleen J. Motil, M.D., Ph.D.

Krupa Mysore, M.D.

Anthony P. Olivé, M.D.

Yen Pham, M.D.

Sarah M. Phillips, M.S., R.D.

Priya Raj, M.D., M.S.

Carol A. Redel, MNS, M.D.

Ann O. Scheimann, M.D.

Vernisha Y. Shepard, M.Ed., LPC

Robert J. Shulman, M.D.

Mary Elizabeth Tessier, MD

Sundararajah Thevananther, Ph.D.

Nicole Triggs,MSN,APRN,CPNP,CPN

Kristin L. Van Buren, M.D.

Bryan S. Vartabedian, M.D.

Seema Mehta Walsh, M.D.

Allyson E. Wyatt, M.D.

##### TELEPHONE

**Office:**

Office Fax:	832-825-3633

Clinic:	832-822-3131

Clinic Fax:	832-825-4131

Appointments:	832-822-2778

OUTREACH CLINICS

Sugar Land	281-494-7010

Cy-Fair	281-469-4688

Clearlake	281-282-1900

West Campus	832-227-1440

Woodlands	936-321-0808

**Response to the Reviewers**

The time and expertise of the reviewer is greatly appreciated. We responded to the valuable recommendation. The corresponding changes in the manuscript are highlighted with Word ‘track changes’.

In general, we would like to underscore that our work is intended to demonstrate the unreliable nature of epidemiological observations in regards to the effects of historical pediatric BCG vaccination protecting adults/elderly against COVID-19, and does NOT intend to evaluate the biologic evidence for BCG vaccination as an acute (i.e. short lived) protectant against the viral infection. Several small adjustments were made to the text to more clearly make this distinction. We feel that our manuscript has been further improved, and PLOS ONE is an outstanding means to communicate our message and hope that the reviewer agrees.

Sincerely,

Richard Kellermayer M.D., Ph.D.

Associate Professor

Director, Pediatric Inflammatory Bowel Disease Program

Baylor College of Medicine, Texas Children's Hospital

6621 Fannin St., CC1010.00

Houston, TX 77030-2399

Voice: 713-798-0319; Fax: 713-798-0338

Reviewer #2: The conclusions from the ongoing clinical trials testing whether BCG could protect against COVID-19 are not yet known, however a recent paper demonstrates that BCG can protect the elderly against respiratory infections (Giamarellos-Bourboulis et al., Cell, 31 Aug 2020). The authors might want to cite this reference as a stronger evidence to test the hypothesis rather than epidemiological data.

Response: We agree, and have added an additional paragraph to the discussion, which critically reviews the recommended publication from our manuscript’s point of view. We also made minor modifications to the paper in order to more clearly emphasize that our work only pertains to the epidemiology of pediatric age delivered, universal BCG vaccination, and whether that may be protective against COVID-19 deaths in the adult/elderly population, who received the vaccine when they were very young (i.e. decades before a possible infection with COVID-19).
